# Fabrication of Highly Photostable Polystyrene Films Embedded with Organometallic Complexes

**DOI:** 10.3390/polym14051024

**Published:** 2022-03-03

**Authors:** Dina S. Ahmed, Alaa Mohammed, Amani A. Husain, Gamal A. El-Hiti, Mohammed Kadhom, Benson M. Kariuki, Emad Yousif

**Affiliations:** 1 Department of Medical Instrumentation Engineering, Al-Mansour University College, Baghdad 64021, Iraq; dina.saadi@muc.edu.iq; 2 Department of Chemistry, College of Science, Al-Nahrain University, Baghdad 64021, Iraq; alaaalqaycy7@gmail.com (A.M.); emad.yousif@ced.nahrainuniv.edu.iq (E.Y.); 3 Polymer Research Unit, College of Science, Al-Mustansiriyah University, Baghdad 10052, Iraq; amani.eyad@uosamarra.edu.iq; 4 Department of Optometry, College of Applied Medical Sciences, King Saud University, Riyadh 11433, Saudi Arabia; 5 Department of Environmental Science, College of Renewable Energy and Environmental Science, Alkarkh University of Science, Baghdad 10081, Iraq; makbq6@mail.missouri.edu; 6 School of Chemistry, Cardiff University, Main Building, Park Place, Cardiff CF10 3AT, UK; kariukib@cardiff.ac.uk

**Keywords:** photodegradation, polystyrene, ibuprofen, organometallic complexes, photostabilization, ultraviolet absorbers

## Abstract

Polystyrene is a common thermoplastic and is produced in different shapes and forms. The scale of manufacture of polystyrene has grown over the years because of its numerous applications and low cost of production. However, it is flammable, brittle, has low resistance to chemicals, and is susceptible to photodegradation on exposure to ultraviolet radiation. There is therefore scope to improve the properties of polystyrene and to extend its useful lifetime. The current work reports the synthesis of organometallic complexes and investigates their use as photostabilizers for polystyrene. The reaction of excess ibuprofen sodium salt and appropriate metal chlorides in boiling methanol gave the corresponding complexes excellent yields. The organometallic complexes (0.5% by weight) were added to polystyrene and homogenous thin films were made. The polystyrene films blended with metal complexes were irradiated with ultraviolet light for extended periods of time and the stabilizing effects of the additives were assessed. The infrared spectroscopy, weight loss, depression in molecular weight, and surface morphology of the irradiated blends containing organometallic complexes were investigated. All the synthesized organometallic complexes acted as photostabilizers for polystyrene. The damage (e.g., formation of small polymeric fragments, decrease in weight and molecular weight, and irregularities in the surface) that took place in the polystyrene blends was much lower in comparison to the pure polystyrene film. The manganese-containing complex was very effective in stabilizing polystyrene and was superior to cobalt and nickel complexes.

## 1. Introduction

Plastic is a very versatile material and has many valuable qualities; it is cheap, light, strong, and can be colored. Plastic is therefore an excellent substitute for glass which is heavy, and wood which is expensive [1]. Polystyrene (PS) is a thermoplastic polymer that has desirable and unique properties suited to many applications. Plastics, and in particular PS, have thus become ubiquitous in everyday life. The usage of PS includes packaging, construction materials, electronic devices, electrical insulators, car parts, kitchen tools, and other areas [2,3,4]. PS can be produced as hard plastic, which can be recyclable, and soft foams, which are nonrecyclable [5]. The arrangement of phenyl moieties controls the shape of the PS [6]. Commercial PS is amorphous with a random arrangement of the phenyl moieties, whereas crystalline PS is produced when phenyl moieties alternate regularly along the polymer chains. Pure PS is rigid, brittle, clear, and colorless. It is also soluble in halogenated solvents, flammable, nonbiodegradable, and has low heat stability. Additionally, it is susceptible to photodegradation and photodecomposition when exposed to ultraviolet (UV) radiation in the presence of oxygen [7,8]. Thus, PS suffers from natural weathering which shortens its lifetime. The main contributing factors to PS weathering are exposure to sunlight and heat, as photodegradation is driven by irradiation in the presence of oxygen [9,10,11]. Photo-oxidation of PS leads to the formation of free radicals causing bond breaking, embrittlement, stiffness, cracking, deterioration of mechanical properties, and discoloration. In addition, photodecomposition leads to the production of various pollutants that are harmful to the environment. It is therefore vital to protect PS against photodegradation through, for example, additives that enhance its photostability, particularly in outdoor applications.

The most common additives for plastics include colorants, plasticizers, flame retardants, and stabilizers [12,13,14]. Additives should be easy and cheap to produce, efficient at a very low concentration, pose no danger to the environment, and not lead to undesirable changes in the physical properties of plastics (e.g., alteration of color). In addition, additives should be chemically stable, involatile, and lead to a homogenous blend with the polymer. Additives can be used as powders, beads, spheres, and flakes. Additives for the reduction of photodecomposition and photooxidation of plastics can act as absorbers for light, quenchers for energy, decomposers for radicals, and antioxidants [15]. Recently, several additives to enable the use of PS in harsh oxygen-rich conditions have been investigated. Examples of the additives include polyphosphates [16], metal complexes [17], Schiff bases [18,19], and aromatics [20,21,22,23,24,25]. Of interest in this work are additives containing aromatic moieties and/or metals in their structures.

Ibuprofen is a medication for the treatment of fever and inflammation [26,27]. It is solid, chemically stable, aromatic, and contains a heteroatom (oxygen). Due to its aromatic ring, the ibuprofen moiety would be expected to act as a UV absorber. In addition, metals are known to act as peroxide radical decomposers due to their acidic nature. Consequently, metal complexes containing the ibuprofen unit are anticipated to act as photostabilizers for polymers. As a continuation of our previous work [18,19,20], we now report for the first time the synthesis of three metallic complexes containing the ibuprofen moiety and investigation of their applicability as stabilizers for PS.

## 2. Materials and Methods

### 2.1. Chemicals and Instrumentation

Polystyrene (*M_W_* = 250,000), ibuprofen (98%), manganese chloride tetrahydrate (MnCl_2_ 4H_2_O; 98%), cobalt chloride hexahydrate (CoCl_2_ 6H_2_O; 98%), and nickel chloride hexahydrate (NiCl_2_ 6H_2_O; 99.9%), were purchased from Merck (Gillingham, UK). The percentage of hydrogen and carbon within the complexes was determined using the Vario EL III analyzer (Elementar Americas, Ronkonkoma, NY, USA). The metal (Mn, Co, and Ni) content within the complexes was determined using an AA-6880 Shimadzu atomic absorption spectrophotometer (Shimadzu, Tokyo, Japan). The Fourier transform infrared (FTIR) spectra (400–4000 cm^−1^) were recorded on an FTIR 8300 Shimadzu spectrophotometer (Shimadzu, Tokyo, Japan). The UV spectra (200–600 nm) were recorded in ethanol at 25 °C on a Shimadzu UV-1601 UV-VIS spectrophotometer (Shimadzu, Tokyo, Japan) using a 1.0 cm quartz cell. No NMR spectra were recorded for the synthesized complexes due to their poor solubility in deuterated solvents. Molar conductivity was measured at 25 °C at a concentration of 1 × 10^−3^ M using a ProfiLine Oxi 3205 conventional portable meter (Xylem Inc., Weinheim, Germany). The magnetic susceptibility was measured using a Bruker BM6 magnetic balance (Bruker, Zürich, Switzerland). The UV irradiation was carried out at 25 °C using a QUV accelerated weather tester (Q-Panel Company; Homestead, FL, USA). The surface morphology of materials was investigated using an FEI Inspect S50 microscope (FEI Company, Czechia, Czech Republic), a Meiji Techno microscope (Tokyo, Japan), and a Veeco system (Plainview, NY, USA).

### 2.2. Synthesis of Metal Complexes

**Scheme 1:**
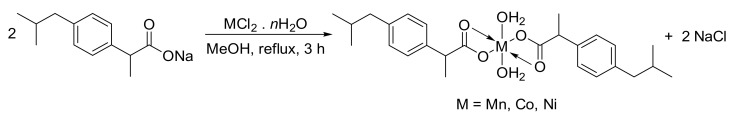
Synthesis of ibuprofen–metal complexes.

A mixture of ibuprofen (0.62 g, 3.0 mmol) and sodium hydroxide (0.12 g, 1.0 mmol) in methanol (MeOH; 25 mL) was stirred for one hour at 25 °C. The solid (ibuprofen sodium salt) produced was filtered, washed with diethyl ether, and dried in the air. A mixture of ibuprofen sodium salt (0.46 g, 2.0 mmol) and appropriate hydrated metal chloride (1.0 mmol) in MeOH (25 mL) was refluxed for 3 h. The solid formed was filtered, washed with MeOH, and dried to give the corresponding ibuprofen–metal complex (Scheme 1) as a powder in excellent yield. The color, melting points (°C), yield (%), and content (%) of carbon, hydrogen, and metal of the synthesized complexes are recorded in Table 1.

### 2.3. Films Preparation and Irradiation

The polymer blends were prepared by mixing PS (10 g) and appropriate ibuprofen-metal complex (50 m) in chloroform (100 mL) at 25 °C. The resulting homogenous solution was stirred for two hours using a magnetic stirrer. The solution was transferred onto a glass slide containing holes (thickness = 40 μm) and left to dry in the air for 6 h. The films produced were dried further in a vacuum oven at 50 °C for 18 h. The PS blends were irradiated at 25 °C for a period ranging from 50 to 300 h using UV light with an intensity of 6.43 × 10^−9^ ein·dm^−3^· s^−1^ and a wavelength (λ_max_) of 365 nm.

## 3. Results and Discussion

### 3.1. Synthesis of Ibuprofen–Metal Complexes

Three metal (Mn, Co, and Ni) complexes containing ibuprofen were synthesized (Scheme 1) with excellent yields (Table 1). The FTIR spectrum for ibuprofen has a broad peak at 3000–3300 cm^−1^ corresponding to the O-H stretching vibrations of the hydroxyl group of the carboxylic acid moiety, which disappeared on formation of the sodium salt and metal complexes. The absence of these vibration bands in the complexes is a clear indication of deprotonation. The asymmetric (ν_asym_) and symmetric (ν_sym_) vibrations corresponding to the carboxylate (COO^−^) moiety in the metal complexes appeared in the 1789–1799 cm^−1^ and 1400–1408 cm^−1^ regions, respectively (Table 2). The differences between the asymmetric and symmetric vibrations of COO^−^ [Δν (asym − sym)] were in the 389–397 cm^−1^ range. Such differences indicated that the coordination between the carboxylate group of ibuprofen and the metal is asymmetry bidentate [28,29]. The FTIR spectra of the synthesized complexes are shown in Figures S1–S3.

The electronic spectral data of the ligand and metal complexes are shown in Table 3. Based on the magnetic susceptibility (μ_eff_), the ibuprofen–metal complexes have octahedral geometry. The UV–vis spectrum of the Mn complex showed absorption bands at 378 nm (26,455 cm^−1^), 523 nm (19,120 cm^−1^), and 642 nm (15,573 cm^−1^), which correspond to the ^6^A_1g_ → ^4^T_2g_(D), ^6^A_1g_ → ^4^T_2g_(G), and ^6^A_1g_ → ^4^T_1g_ transitions, respectively. The Mn complex had a sp^3^d^2^ high spin hybridization with a μ_eff_ of 5.9 [30]. The electronic spectrum of the Co complex displayed absorption bands at 343 nm (29,155 cm^−1^) and 562 nm (17,794 cm^−1^), due to ^4^T_1_g(F) → ^4^A_2_g(F) and ^4^T_1_g(F) → ^4^T_1_g(P) transitions, respectively. The Co complex had a sp^3^d^2^ high spin hybridization with a μ_eff_ of 4.5 BM [30]. For the Ni complex, the electronic spectrum showed absorption bands at 339 nm (29,499 cm^−1^) and 417 nm (23,981 cm^−1^), due to the of ^3^A_2_g(F) → ^3^T_1_g(P) and ^3^A_2_g(F) → ^3^T_1_g(F) transitions, respectively. The μ_eff_ of the Ni complex was 3.1 BM with a sp^3^d^2^ high spin hybridization [31]. The molar conductivity (Λ_m_) was low (0–10 µS/cm; Table 3), which indicated that the synthesized metal complexes behaved as nonelectrolytes [32]. The UV–vis spectra of the synthesized complexes are shown in Figures S4–S6.

**Figure 1:**
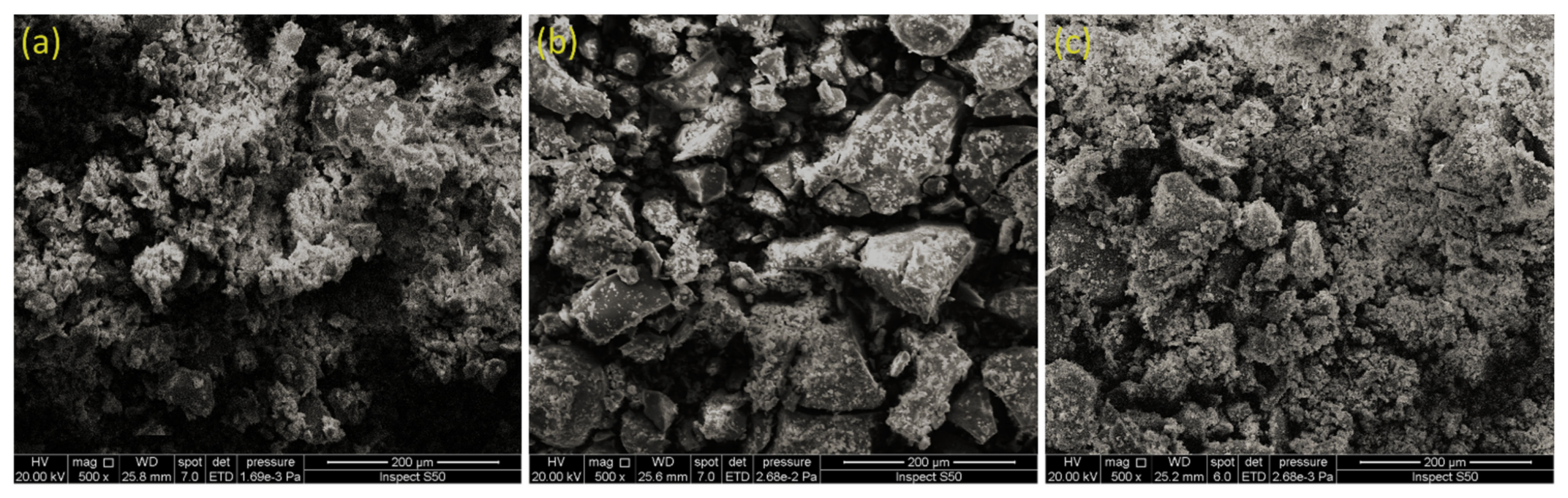
SEM images of (**a**) ibuprofen–Mn complex, (**b**) ibuprofen–Co complex, and (**c**) ibuprofen–Ni complex.

The nature of the surface of the metal complexes was inspected by scanning electron microscopy (SEM) [33]. The SEM images of the synthesized ibuprofen–metal complexes (Figure 1) showed agglomerates and homogenous surface. The particles had irregular morphology with diameters that ranged from 60 to 950 nm. It should be noted that the SEM images of the complexes indicated that the materials produced did not contain metal oxides or hydroxides.

### 3.2. Investigation of Photostability of PS Using FTIR Spectrometry

**Figure 2:**
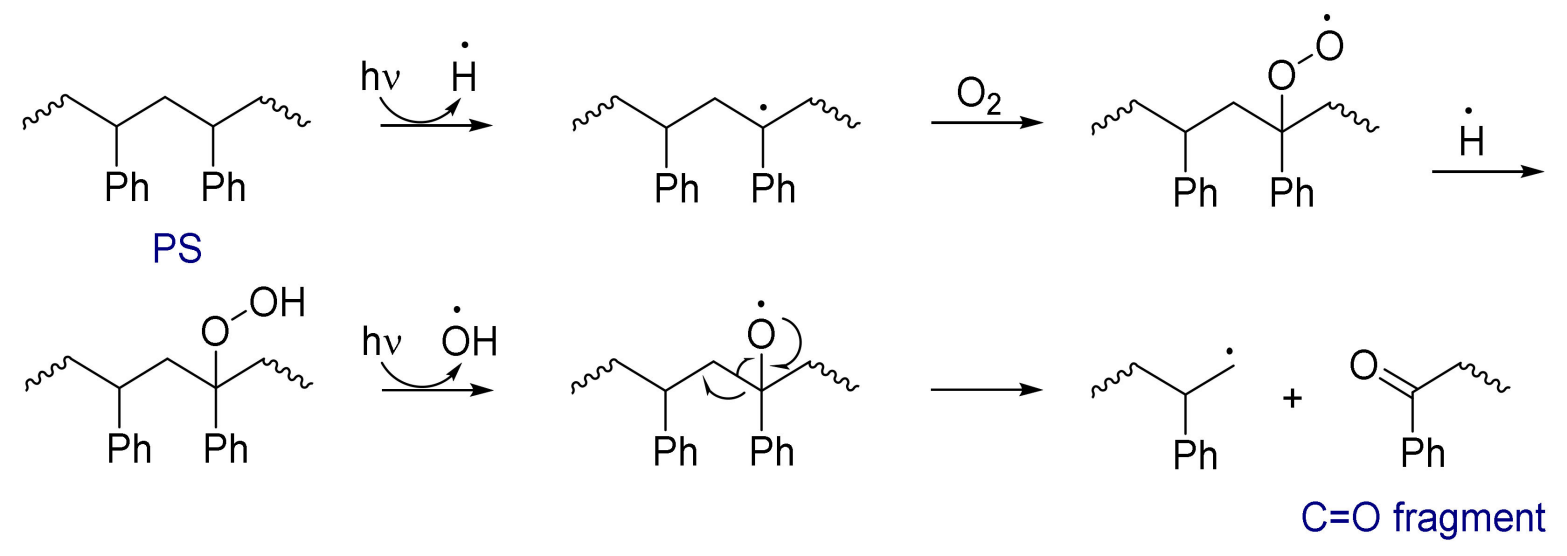
Photodegradation of PS in the presence of oxygen leading to formation of carbonyl group-containing fragments.

The irradiation of PS causes a loss of mechanical properties, discoloration, and the formation of small polymeric fragments that contain various functional groups [34,35,36]. An example is the irradiation of PS causing the elimination of hydrogen radicals (Figure 2). In the presence of oxygen, the PS radicals produced lead to the formation of very reactive oxygenated species, which can combine with hydrogen radicals to generate hydroxylated PS. The hydroxylated PS splits into hydroxyl radicals and oxygenated PS radicals, that finally yield carbonyl group-containing fragments (Figure 2). Monitoring of the –C=O group by FTIR spectroscopy on irradiation of PS blends is a source of important information about the level of photodegradation.

**Figure 3:**
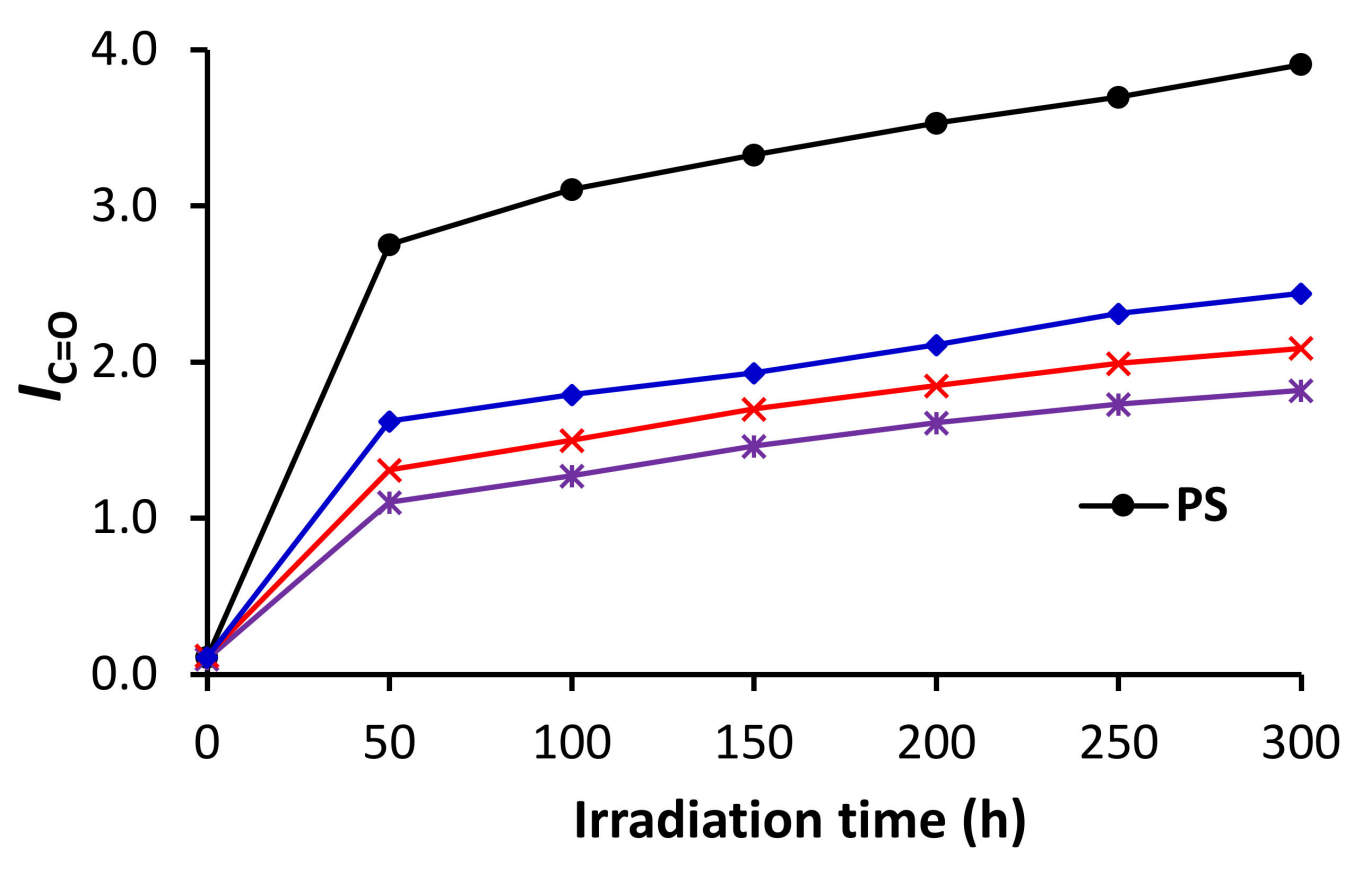
A plot of I_C=O_ of PS films as a function of UV irradiation time.

Therefore, the effect of the ibuprofen–metal complexes on the photodegradation of PS was investigated using FTIR spectrometry [37,38]. The pure PS and PS blends were separately exposed to UV radiation for 300 h with samples being taken for analysis every 50 h. The intensity (A_C=O_) of the –C=O (1720 cm^−1^) band, which increased due to the formation of carbonyl group-containing fragments, was monitored and compared with that for the –C–H band (A_C-H_) of the CH_2_ groups, which are not affected by irradiation [9,39]. The increase in the –C=O group index (I_C=O_) was estimated using Equation (1) and plotted as a function of the irradiation time (Figure 3).

(1) $$I_{C=O}=\frac{A_{C=O}}{A_{C-H}}$$

Figure 3 shows that the increase in the *I*_C=O_ was fast and sharp in the first 50 h and then continued at a slower but steady rate after that. The addition of ibuprofen–metal complexes led to a noticeable reduction in the *I*_C=O_, reflecting their abilities to reduce cleavage of the polymeric chains and subsequent formation of small fragments containing carbonyl groups. The I_C=O_ was 2.75, 1.10, 1.31, and 1.62 for the pure PS and those containing Mn, Co, and Ni complexes after 50 h of irradiation, respectively. At the end of the irradiation process, the I_C=O_ was 3.91 for the pure PS film and 1.82, 2.09, and 2.44 for the blends containing Mn, Co, and Ni complexes, respectively. Clearly, the complex containing Mn led to the highest reduction of photodegradation of PS, with Co and Ni complexes close behind.

### 3.3. Investigation of Photostability of PS Using Weight Loss Analysis

**Figure 4:**
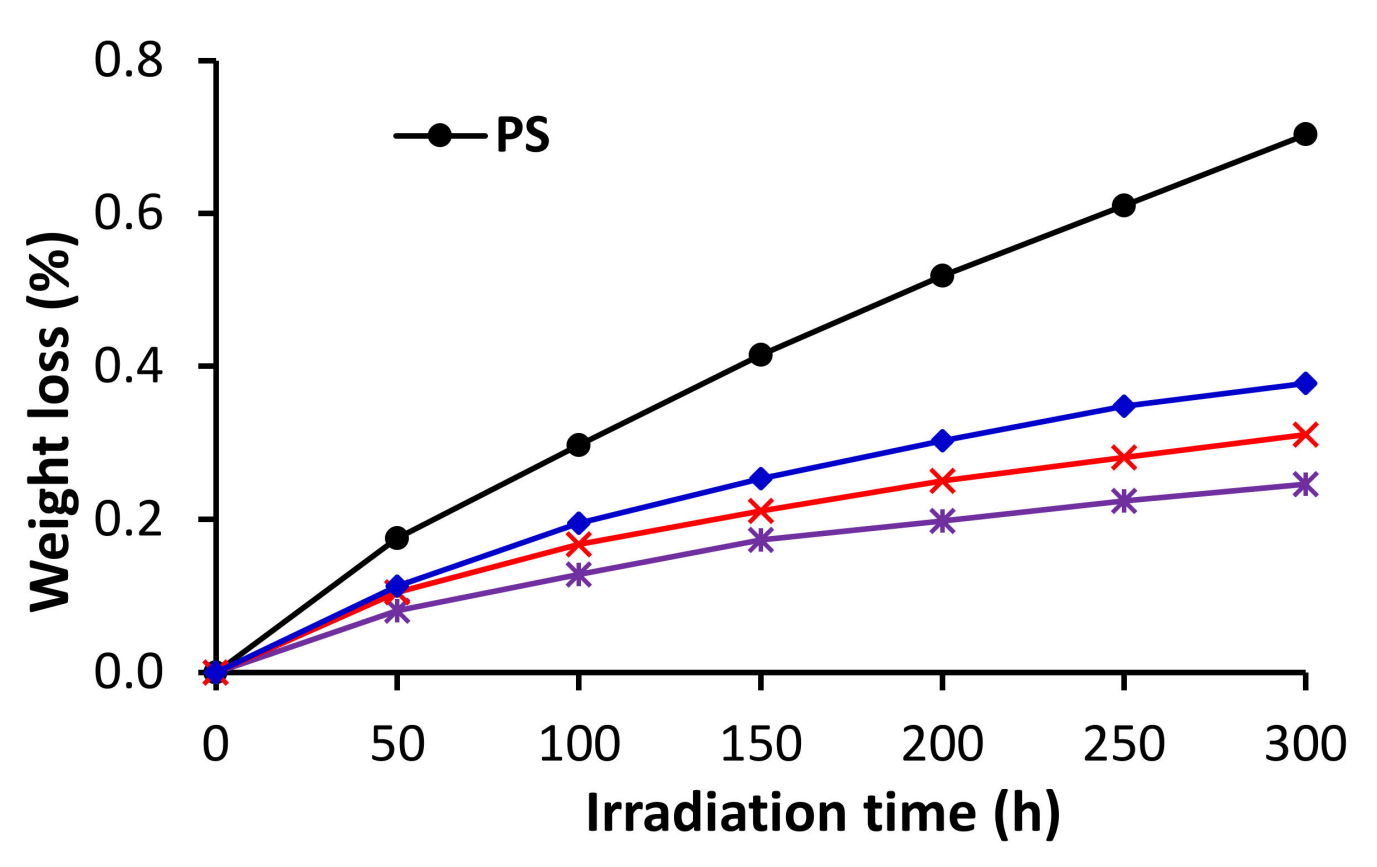
Weight loss (%) of PS films as a function of UV irradiation time.

Photooxidation and photodegradation of PS lead to the formation of free radical species, bond breaking, and cross-linking. These processes can produce volatile small molecular weight residues resulting in weight loss [10,24]. The weight of pure PS and the blends was measured before (*W_0_*) and after (*W_t_*) irradiation, and the percentage of the weight loss was determined using Equation (2) and plotted as a function of time (Figure 4).

(2) $$\mathrm{Weight}\;\mathrm{loss}\;\left(\%\right)=\frac{W_{0}-W_{t}}{W_{0}}\times 100$$

Figure 4 shows that the weight loss percentage was highest in the case of the pure PS film. The ibuprofen–metal complexes reduced the weight loss noticeably and the Mn complex was the most efficient additive at inhibiting PS photodegradation. The weight loss was sharpest at the beginning of the irradiation and continued throughout the experiment. After 300 h, the weight loss was 0.70, 0.25, 0.31, and 0.38% for the pure PS film and the blends containing Mn, Co, and Ni complexes, respectively.

### 3.4. Investigation of Photostability of PS Using Molecular Weight

**Figure 5:**
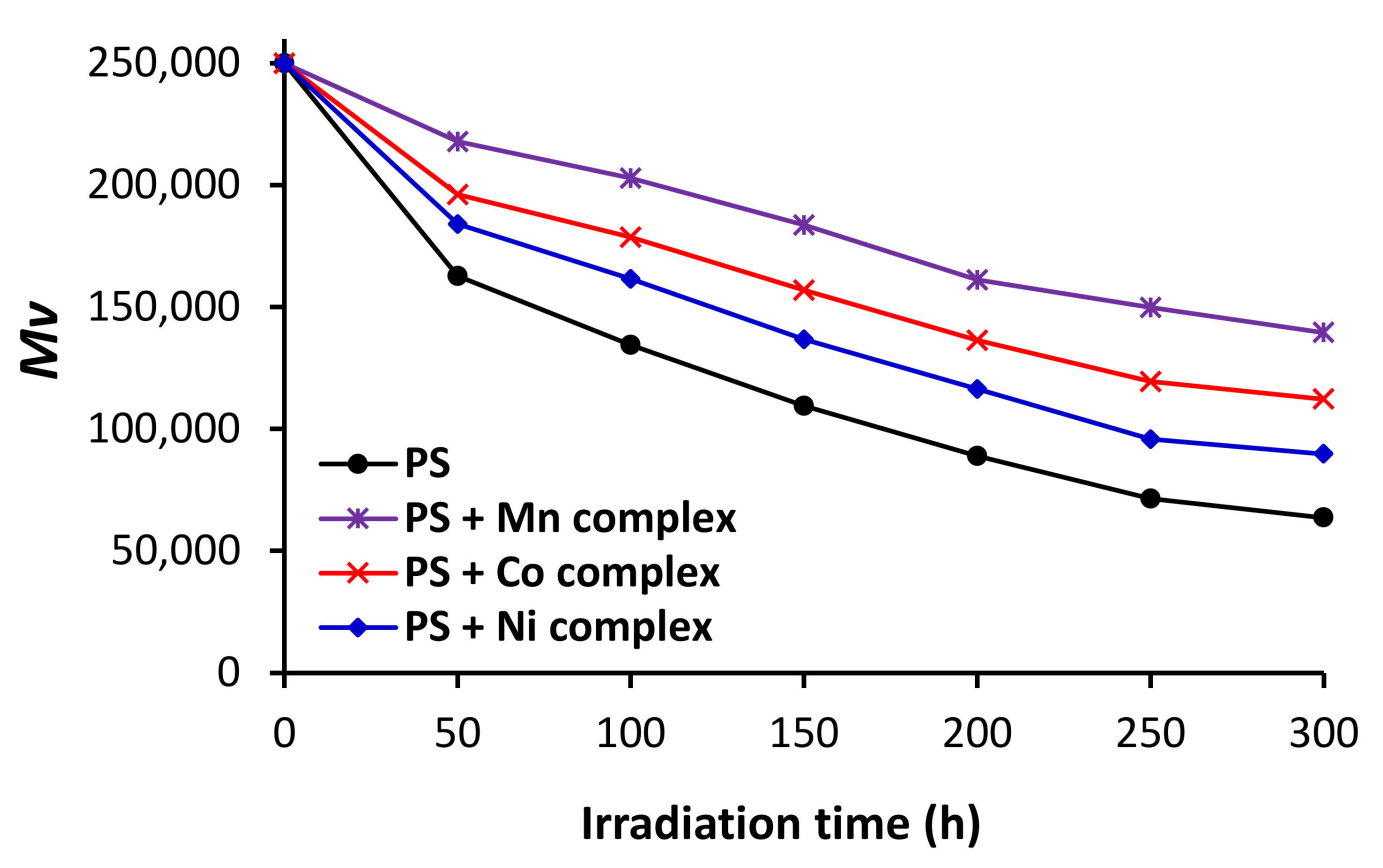
Changes in the $\bar{M}v$ of PS films as a function of UV irradiation.

There is an inverse relationship between the time of irradiation and the PS average molecular weight ($\bar{M}v$.) [18,40]. Photodegradation of PS leads to a decrease in the $\bar{M}v$ due to bond breaking, cross-linking, and formation of small fragments that have low molecular weight. Thus, the damage caused within the PS could be evaluated by observation of the reduction in the $\bar{M}v$ as a function of irradiation time. Therefore, the PS blends were irradiated for 300 h, and samples taken at 50 h intervals were dissolved in tetrahydrofuran and the intrinsic viscosity [*η*] of the solution measured. The $\bar{M}v$ was calculated as a function of [*η*] using Equation (3) [41]. Figure 5 shows the effect of UV irradiation on the $\bar{M}v$ of the PS blends in the presence and absence of ibuprofen–metal complexes.

(3) $$\left[\eta \right]=1.63\times 10^{-2}\;M_{v}^{0.766}$$

The decrease in the $\bar{M}v$ of the PS blends was most significant in the absence of ibuprofen–metal complexes, as indicated by $\bar{M}v$ decreases from 250,000 to 162,723, 88,996, and 63,714 after 50,200, and 300 h of irradiation, respectively. Notably, the pure PS film lost around 75% of its $\bar{M}v$. by the end of the irradiation process. On the other hand, the decrease in the $\bar{M}v$. in the presence of ibuprofen–metal complexes was much less compared with the pure film. Particularly effective was the Mn complex, with $\bar{M}v$ values of 217,942, 161,207, and 139,564 after 50, 200, and 300 h of irradiation of the PS blend, respectively.

### 3.5. Investigation of Photostability of PS Using Surface Morphology

The effect of ibuprofen–metal complexes was investigated through the inspection of the surfaces of the irradiated blends. The surface morphology of the PS can provide valuable information about irregularities (e.g., spots, grooves, cracks, and darkness) that can result from the photodegradation of PS. An optical microscope, which can be very informative in the study surfaces, was used first [42,43].

**Figure 6:**
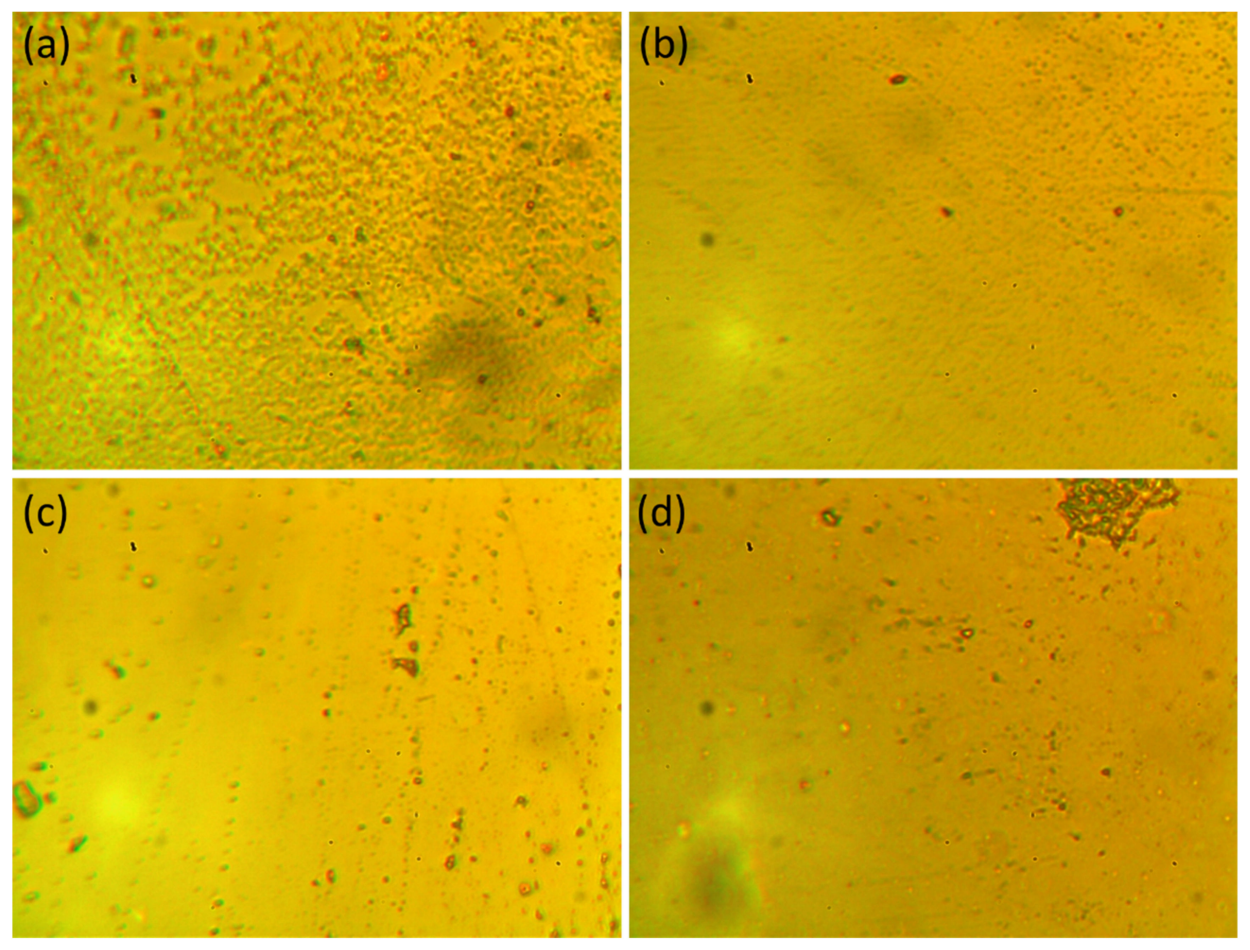
Microscope images recorded after 300 h of UV irradiation of PS blends containing (**a**) no additives, (**b**) ibuprofen–Mn complex, (**c**) ibuprofen–Co complex, and (**d**) ibuprofen–Ni complex.

Figure 6 shows that, after irradiation, the surface of the pure PS film was rough and contained a larger number of cracks and dark spots when compared to the blends containing the ibuprofen–metal complexes. The surface irregularity is mainly due to cross-linking and chain scission that occurred because of PS photodegradation [44]. It is clear that the presence of ibuprofen–metal complexes protected the PS against photodegradation. The surface of the PS containing the Mn complex was smoothest and contained the lowest number of defects compared to the others.

**Figure 7:**
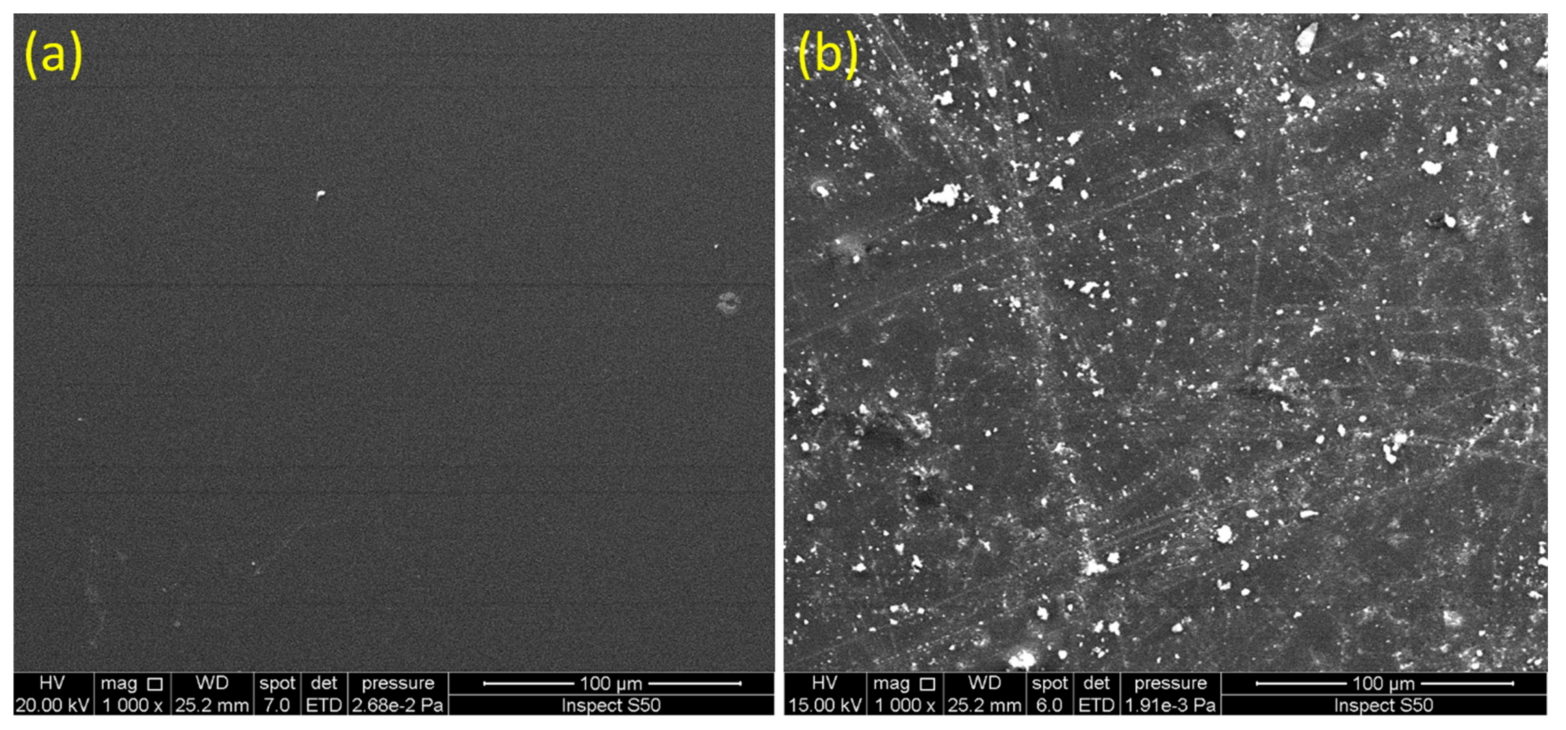
SEM images of pure PS film (**a**) before irradiation and (**b**) after 300 h of UV irradiation.

**Figure 8:**
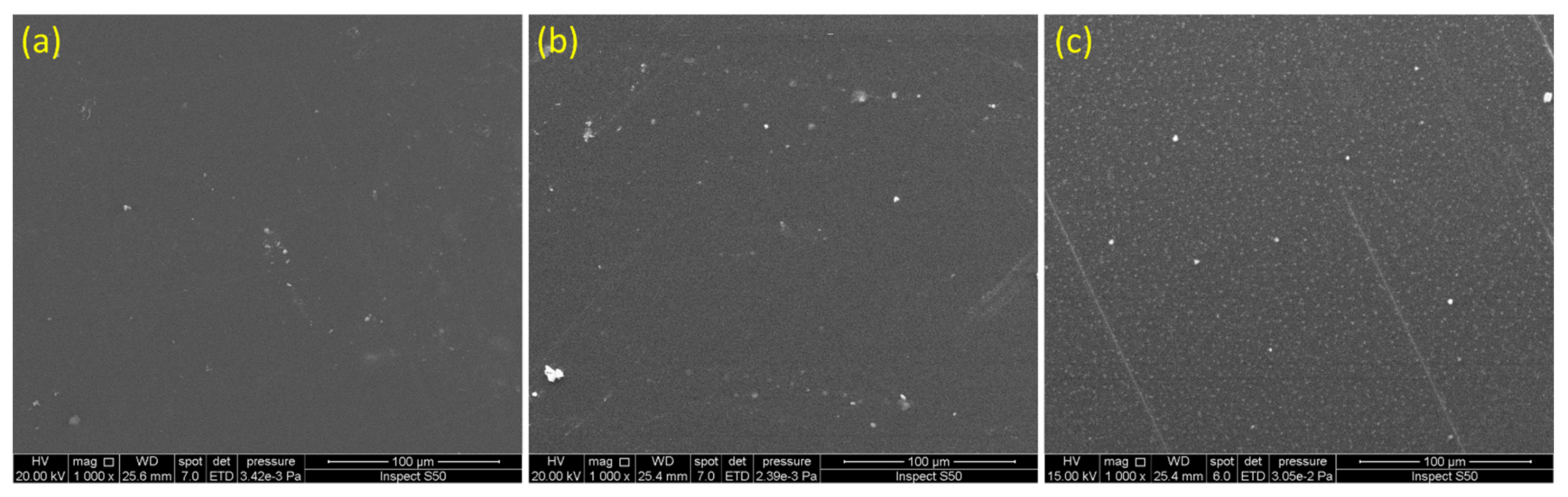
SEM images recorded after 300 h of irradiation of PS films containing (**a**) ibuprofen–Mn complex, (**b**) ibuprofen–Co complex, and (**c**) ibuprofen–Ni complex.

The SEM technique is a valuable tool for inspection of the PS surfaces after UV irradiation [45,46]. The SEM images of the pure PS film, before and after UV irradiation, are shown in Figure 7. It was clear the surface of the nonirradiated PS film was homogenous and smooth with no defects (e.g., spots or grooves). In contrast, the surface of the irradiated PS film showed a high number of white spots which resulted from photodegradation. On the other hand, the surfaces of the irradiated PS blends containing the ibuprofen–metal complexes showed far fewer white spots, consistent with their abilities to protect PS against photodegradation (Figure 8).

**Figure 9:**
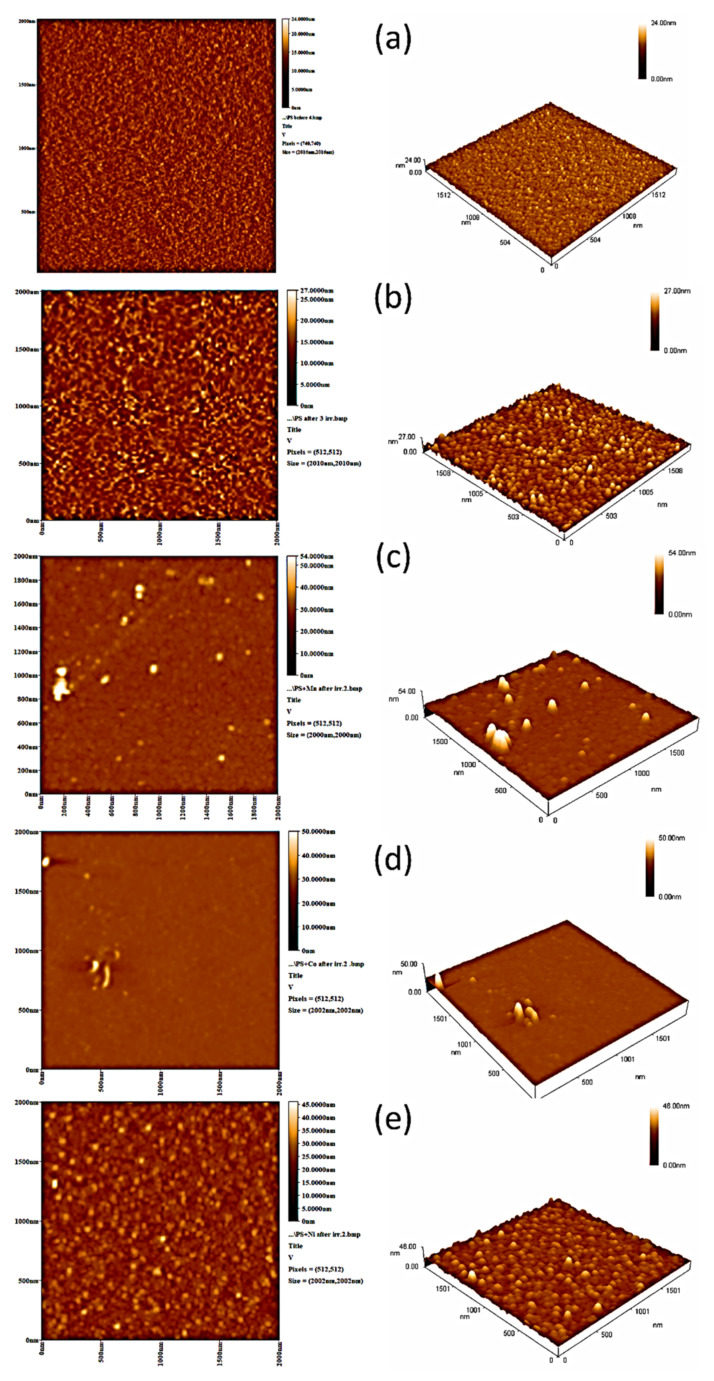
AFM images of (**a**) PS film before irradiation and after irradiation of (**b**) pure PS film, (**c**) PS + ibuprofen–Mn complex, (**d**) PS + ibuprofen–Co complex, and (**e**) PS + ibuprofen–Ni complex.

Atomic force microscopy (AFM) was also used to inspect the surface of the polymeric blends to evaluate the level of damage resulting from photodegradation [47,48,49,50]. The AFM image of the PS film before irradiation showed a smooth and regular surface (Figure 9a). In the absence of the additives, the AFM image of the irradiated PS film showed imperfection with the appearance of irregular regions (Figure 9b). In the presence of the ibuprofen–metal complexes, the dark spots were much fewer than those observed for the pure PS film (Figure 9c–e). These results are farther evidence that the ibuprofen–metal complexes successfully acted as photostabilizers.

The roughness factor (*Rq*) is a measure of the smoothness of the surface of materials. The ibuprofen–metal complexes led to a reduction in the *Rq* of the PS and showed better performance than a range of additives used recently to stabilize polyvinyl chloride [51]. The *Rq* was ca. 6.5 for the nonirradiated pure PS film. The *Rq* values for the irradiated PS blends were 340.2, 25.8, 63.7, and 75.3 for the pure PS film and the blends containing the Mn, Co, and Ni complexes, respectively. It was clear that, at 13.2-fold, the ibuprofen–Mn complex led to the most reduction in the *Rq* (Table 4). Clearly, the ibuprofen–Mn complex is more effective in stabilizing PS compared with Schiff bases of both biphenyl-3,3′,4,4′-tetraamine [18] and cephalexin [19], possibly due to the acidic character of the Mn atom that enables the complex to be a better radical scavenger. On the other hand, the 1,2,3,4-triazole-3-thiol Schiff bases [20] showed a better performance than the synthesized metal complexes, due to their high contents of heteroatoms and aromatic moieties.

### 3.6. Proposed Mechanisms for PS Photostability Using Ibuprofen–Metal Complexes

**Scheme 2:**
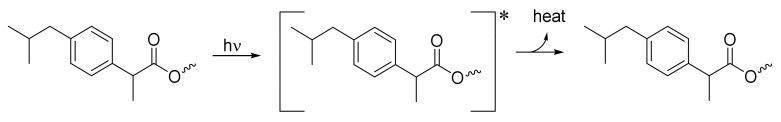
Ibuprofen–metal complexes as UV light absorbers. ٭ Represents the excited state of the intermediate.

The synthesized ibuprofen–metal complexes have been proven to be effective photostabilizers for PS against UV irradiation. The additives contain an aromatic moiety that can absorb UV light efficiently (Scheme 2). The absorbed light can then be released as heat at a rate that is not harmful to the PS chains [52,53].

**Scheme 3:**
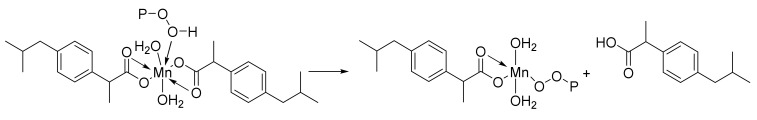
Ibuprofen–Mn complex acts as a hydroperoxide decomposer.

Of the synthesized materials, the ibuprofen–Mn complex has the largest, most easily accessible cation, and is therefore the most capable of decomposing the hydroperoxide (POOH) responsible for PS photodecomposition. Thus, the additive acts as a hydroperoxide decomposer (Scheme 3) [17].

**Scheme 4:**
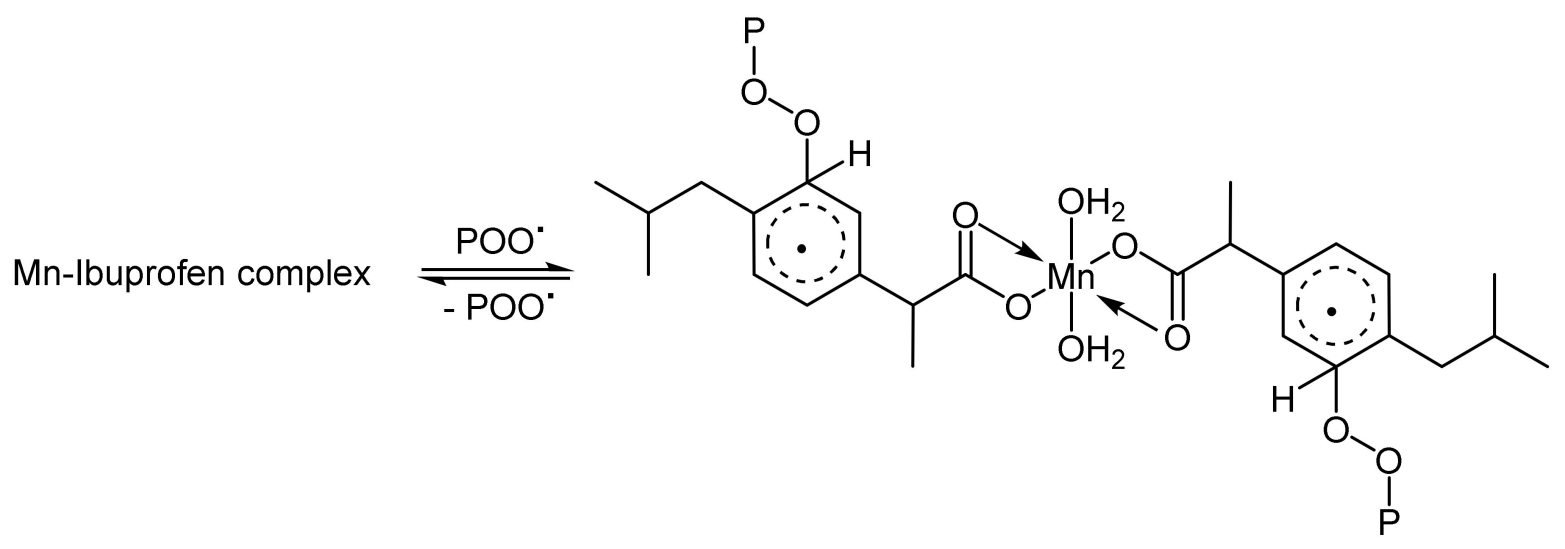
Ibuprofen–Mn complex as a peroxide radical scavenger.

In addition, the ibuprofen–metal complexes, and in particular the complex containing Mn are radical scavengers. They react with peroxide radicals (POO^.^) and act as chromophores leading to the production of highly stable intermediates due to aromatic moiety resonance (Scheme 4) [17].

## 4. Conclusions

Three metal complexes containing the ibuprofen moiety were synthesized with excellent yields using a simple procedure. Ultraviolet and infrared spectroscopy were performed, and the elemental composition, magnetic susceptibilities, and molar conductivities determined for the synthesized metal complexes. The metal coordination in the complexes was octahedral in geometry. The metal complexes were mixed with polystyrene to explore their effect on the photostability of the polymeric blends. As anticipated, the complexes significantly reduced the photodegradation of polystyrene on irradiation with ultraviolet light. Various techniques, namely infrared spectroscopy, weight loss analysis, decrease in molecular weight, and changes in the surface of polystyrene were used to explore the stabilizing effect of the synthesized ibuprofen–metal complexes. The metal complexes acted as ultraviolet absorbers, hydroperoxide decomposers, and free radical scavengers. The manganese complex had the most stabilizing effect, relative to cobalt and nickel, as it had the largest active site. It therefore was more capable of binding active species such as peroxides leading to more effective deactivation. The synthesized organometallic complexes were determined to be more effective PS stabilizers than biphenyl-3,3′,4,4′-tetraamine and 1,2,3,4-triazole-3-thiol Schiff bases.

## Figures and Tables

**Table 1:** Physical properties and elemental content of ibuprofen–metal complexes obtained according to Scheme 1.

Complex	Color	M.P. (°C)	Yield (%)	Element Analysis (Calculated; %)		
				C	H	M
Mn	Dark brown	173–175	88	62.36 (62.27)	7.58 (7.64)	11.15 (10.95)
Co	Brown	151–153	90	61.65 (61.78)	7.42 (7.58)	11.53 (11.66)
Ni	Light green	300 (decomp)	91	61.98 (61.78)	7.50 (7.58)	11.49 (11.62)

**Table 2:** FTIR spectral data for the ligand in the ibuprofen sodium salt and metal complexes obtained according to Scheme 1.

Metal Complex	FTIR, Frequency (*ν*, cm^−1^)				
	Carboxylate Group (COO^−^)			C=C	M–O
	asym	sym	Δν (asym − sym)		
Ibuprofen sodium salt	1697	1411	286	1551	—
Mn	1799	1408	391	1558	416
Co	1789	1400	389	1593	428
Ni	1797	1400	397	1589	425

**Table 3:** Electronic spectral data, conductivity, magnetic moments, geometry, and hybridization of ibuprofen sodium salt and metal complexes obtained according to Scheme 1.

Complex	λ (nm)	Absorption (cm^−1^)	Transition	Λ_m_ (µS/cm)	μ_eff_	Geometry	Hybridization
Ibuprofen sodium salt	294	34,014	π → π٭	—	—	—	—
	298	33,557	π → π٭				
Ibuprofen-Mn	300	33,333	π → π٭	10	5.9	Octahedral	sp^3^d^2^ high spin
	315	31,746	π → π٭				
	378	26,455	^6^A_1_g → ^4^T_2_g (D)				
	523	19,120	^6^A_1_g → ^4^T_2_g (G)				
	642	15,573	^6^A_1_g → ^4^T_1_g				
Ibuprofen-Co	295	33,898	π → π٭	10	4.5	Octahedral	sp^3^d^2^ high spin
	304	32,895	π → π٭				
	343	29,155	^4^T_1_g(F) → ^4^A_2_g(F)				
	562	17,794	^4^T_1_g(F) → ^4^T_1_g(P)				
Ibuprofen-Ni	295	33,898	π → π٭	0	3.1	Octahedral	sp^3^d^2^ high spin
	303	33,003	π → π٭				
	339	29,499	^3^A_2_g (F) → ^3^T_1_g (P)				
	417	23,981	^3^A_2_g (F) → ^3^T_1_g (F)				

**Table 4:** The decrease in the *Rq* (by fold) for the irradiated PS blended with different additives.

**PS Additive**	**Reduction in *Rq* (by Fold)**	**Reference**
Ibuprofen–Mn complex	13.2	Current work
Biphenyl-3,3′,4,4′-tetraamine Schiff bases	8.3	[18]
Cephalexin Schiff bases	27.1	[19]
1,2,3,4-Triazole-3-thiol Schiff bases	3.3	[20]

## Data Availability

Data are contained within the article.

## Supplementary Materials

The following supporting information can be downloaded at: https://www.mdpi.com/article/10.3390/polym14051024/s1, Figure S1: FTIR spectrum of ibuprofen–Mn complex, Figure S2: FTIR spectrum of ibuprofen–Co complex, Figure S3: FTIR spectrum of ibuprofen–Ni complex, Figure S4: UV–vis spectrum of ibuprofen–Mn complex, Figure S5: UV–vis spectrum of ibuprofen–Co complex, and Figure S6: UV–vis spectrum of ibuprofen–Ni complex.
