# Supplementary material for: Fabrication of Highly Photostable Polystyrene Films Embedded with Organometallic Complexes
Source: Polymers (Basel). 2022 Mar 3;14(5):1024. doi: 10.3390/polym14051024 (PMC8914741; doi:10.3390/polym14051024)
Supplement: Supplementary file 1 [file polymers-14-01024-s001.zip › polymers-1604018-supplementary.pdf]

polymers

Supplementary Materials

Fabrication of Highly Photostable Polystyrene Films Embedded with Organometallic Complexes

Dina S. Ahmed [1], Alaa Mohammed [2], Amani A. Husain [3], Gamal A. El-Hiti [4,*], Mohammed Kadhom [5], Benson M. Kariuki [6] and Emad Yousif [2]

1 Department of Medical Instrumentation Engineering, Al-Mansour University College, Baghdad 64021, Iraq; dina.saadi@muc.edu.iq
2 Department of Chemistry, College of Science, Al-Nahrain University, Baghdad 64021, Iraq; alaaalqaycy7@gmail.com (A.M.); emad.yousif@ced.nahrainuniv.edu.iq (E.Y.)
3 Polymer Research Unit, College of Science, Al-Mustansiriyah University, Baghdad 10052, Iraq; amani.eyad@uosamarra.edu.iq
4 Department of Optometry, College of Applied Medical Sciences, King Saud University, Riyadh 11433, Saudi Arabia
5 Department of Environmental Science, College of Renewable Energy and Environmental Science, Alkarkh University of Science, Baghdad 10081, Iraq; makbq6@mail.missouri.edu
6 School of Chemistry, Cardiff University, Main Building, Park Place, Cardiff, CF10 3AT, UK; kariukib@cardiff.ac.uk
* Correspondence: gelhiti@ksu.edu.sa; Tel.: +966-11469-3778; Fax: +966-11469-3536

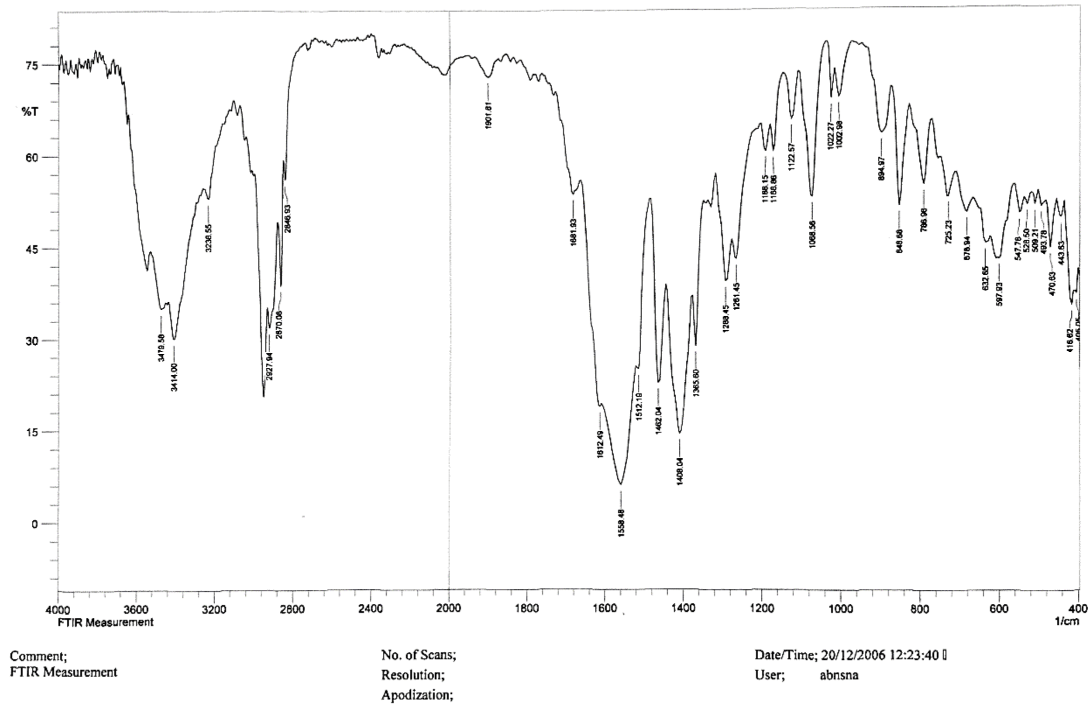

Figure S1. FTIR spectrum of ibuprofen-Mn complex.

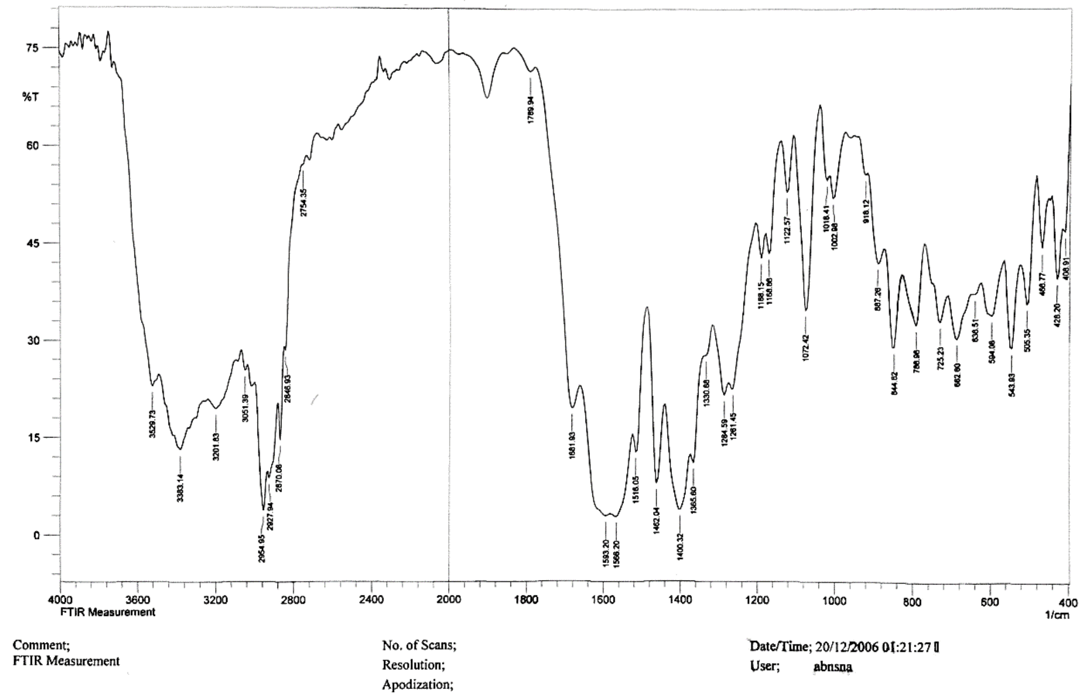

Figure S2. FTIR spectrum of ibuprofen-Co complex.

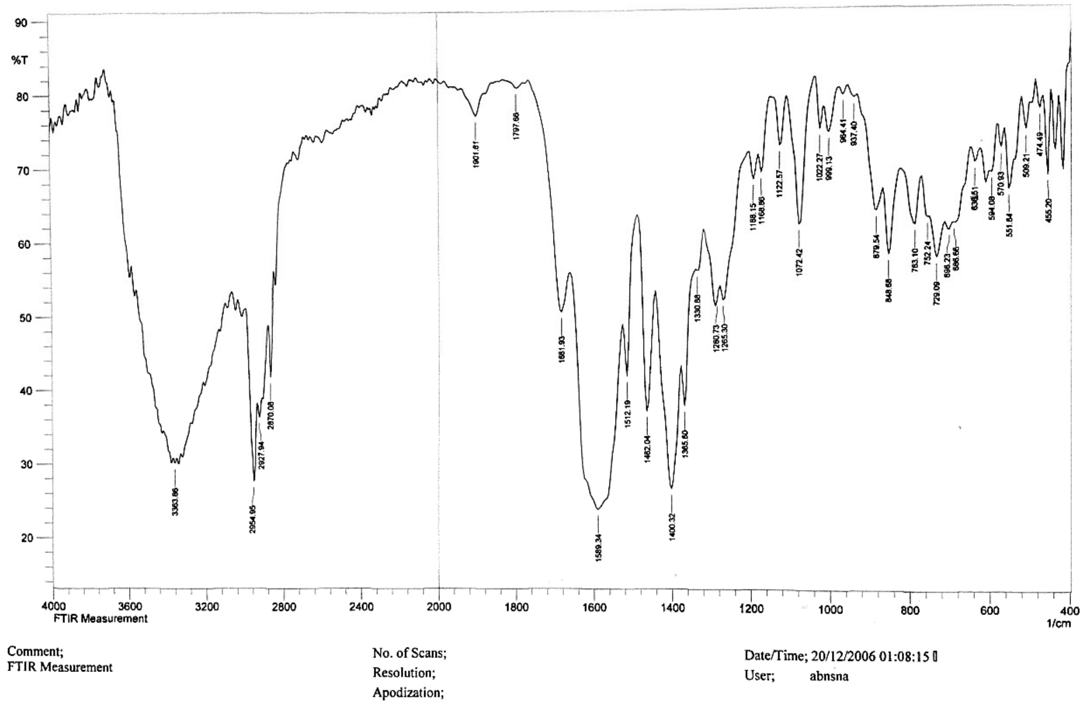

Figure S3. FTIR spectrum of ibuprofen-Ni complex.

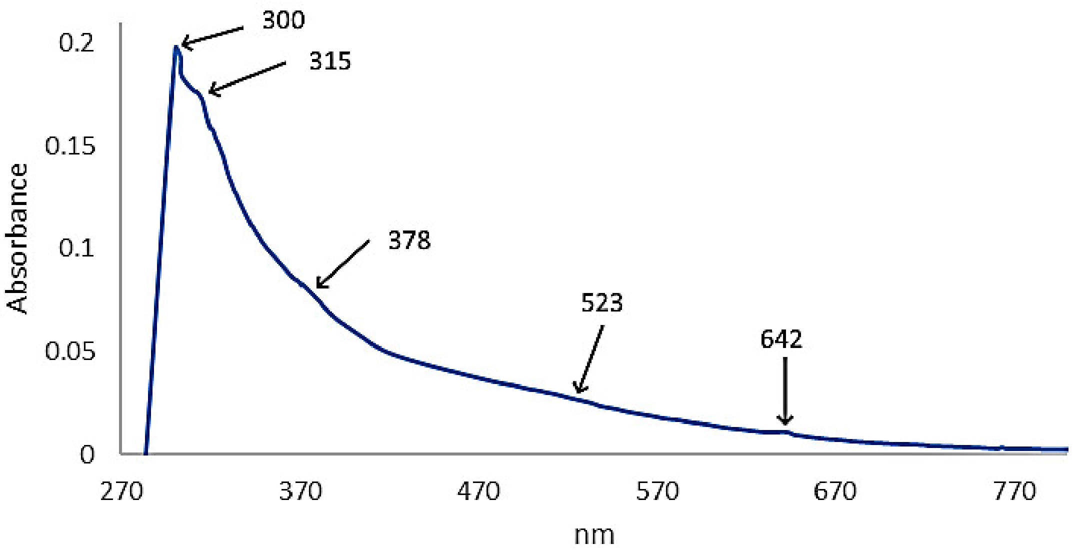

Figure S4. UV-visible spectrum of ibuprofen-Mn complex.

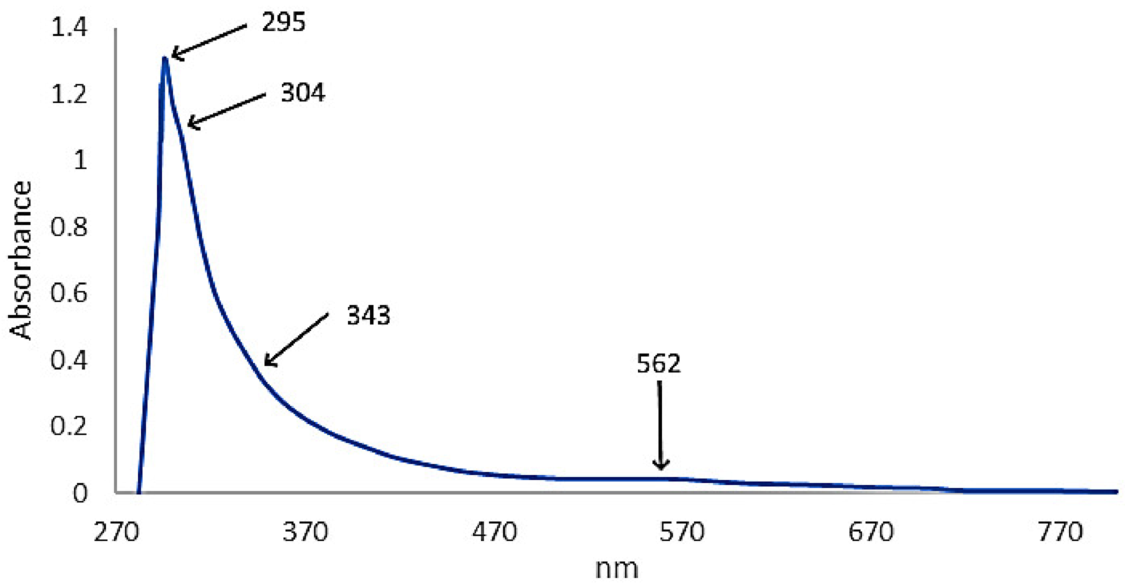

Figure S5. UV-visible spectrum of ibuprofen-Co complex.

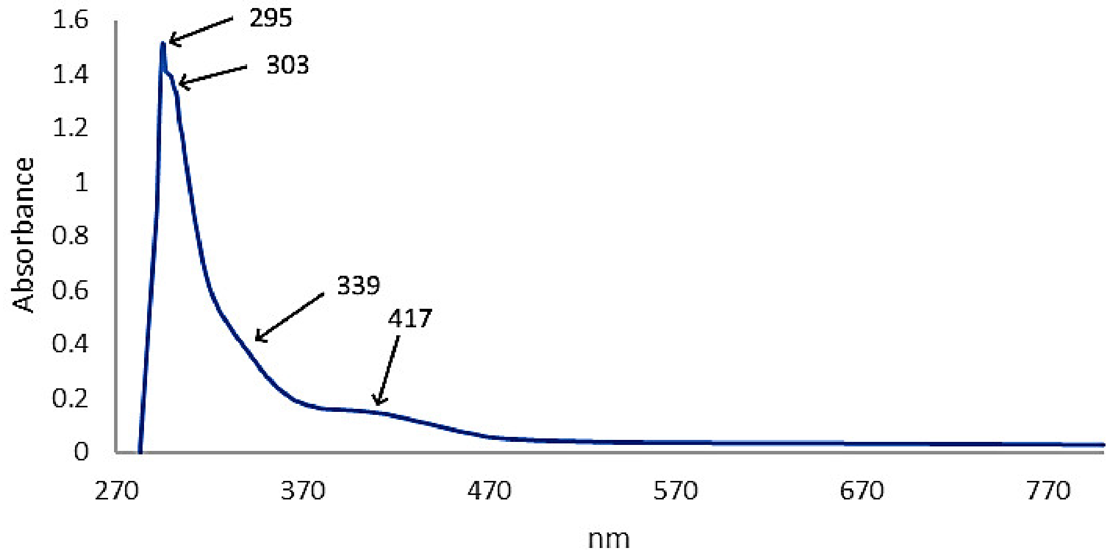

Figure S6. UV-visible spectrum of n ibuprofen-Ni complex.
